# Ethyl 4-(3-bromo­phen­yl)-6-methyl-2-oxo-1,2,3,4-tetra­hydro­pyrimidine-5-carboxyl­ate

**DOI:** 10.1107/S1600536810049019

**Published:** 2010-11-27

**Authors:** Haldorai Yuvaraj, S. Sundaramoorthy, D. Velmurugan, Rajesh G. Kalkhambkar

**Affiliations:** a School of Display and Chemical Engineering, Yeungnam University, Gyeongsan, Gyeongbuk 712-749, Republic of Korea; b Centre of Advanced Study in Crystallography and Biophysics, University of Madras, Guindy Campus, Chennai 600 025, India; c Department of Chemistry, Karnatak Universitys Karnatak Science College, Dharwad 580 001, Karnataka, India

## Abstract

In the title compound, C_14_H_15_BrN_2_O_3_, the dihydro­pyrimidin­one ring adopts a boat conformation. In the crystal, adjacent mol­ecules are linked through N—H⋯O hydrogen bonds forming an *R_2_^2^*(8) ring motif and generating a zigzag chain extending in [010].

## Related literature

For general background to and the pharmaceutical applications of pyrimidino­nes, see: Biginelli (1891 ▶); Atwal (1990 ▶); Kappe (2000 ▶). For a related structure, see: Fun *et al.* (2009 ▶). For ring conformations, see: Cremer & Pople (1975 ▶). For hydrogen-bond motifs, see: Bernstein *et al.* (1995 ▶).
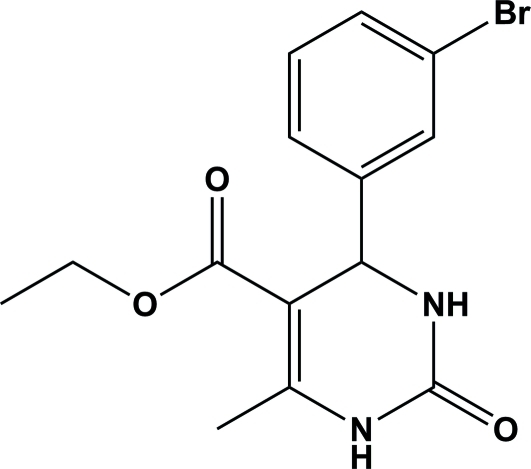


## Experimental

### 

#### Crystal data

C_14_H_15_BrN_2_O_3_
*M* *_r_* = 339.19
Monoclinic, 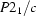

*a* = 12.5184 (11) Å
*b* = 7.3412 (5) Å
*c* = 17.0426 (15) Å
β = 115.086 (6)°
*V* = 1418.5 (2) Å^3^
*Z* = 4
Mo *K*α radiation
μ = 2.91 mm^−1^
*T* = 293 K
0.25 × 0.23 × 0.2 mm

#### Data collection

Bruker SMART APEXII area-detector diffractometer
Absorption correction: multi-scan (*SADABS*; Bruker, 2008 ▶) *T*_min_ = 0.488, *T*_max_ = 0.559
13419 measured reflections
3541 independent reflections
2597 reflections with *I* > 2σ(*I*)
*R*_int_ = 0.033

#### Refinement

*R*[*F* ^2^ > 2σ(*F* ^2^)] = 0.034
*wR*(*F* ^2^) = 0.092
*S* = 1.02
3541 reflections
183 parameters
H-atom parameters constrained
Δρ_max_ = 0.32 e Å^−3^
Δρ_min_ = −0.57 e Å^−3^

### 

Data collection: *APEX2* (Bruker, 2008 ▶); cell refinement: *SAINT* (Bruker, 2008 ▶); data reduction: *SAINT*; program(s) used to solve structure: *SHELXS97* (Sheldrick, 2008 ▶); program(s) used to refine structure: *SHELXL97* (Sheldrick, 2008 ▶); molecular graphics: *ORTEP-3* (Farrugia, 1997 ▶); software used to prepare material for publication: *SHELXL97* and *PLATON* (Spek, 2009 ▶).

## Supplementary Material

Crystal structure: contains datablocks global, I. DOI: 10.1107/S1600536810049019/bt5419sup1.cif

Structure factors: contains datablocks I. DOI: 10.1107/S1600536810049019/bt5419Isup2.hkl

Additional supplementary materials:  crystallographic information; 3D view; checkCIF report

## Figures and Tables

**Table 1:** Hydrogen-bond geometry (Å, °)

*D*—H⋯*A*	*D*—H	H⋯*A*	*D*⋯*A*	*D*—H⋯*A*
N1—H1*A*⋯O1^i^	0.86	2.04	2.868 (2)	161
N2—H2*A*⋯O1^ii^	0.86	2.12	2.948 (2)	162

Symmetry codes: (i) 
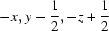
; (ii) 
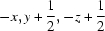
.

**Fig. 1.**
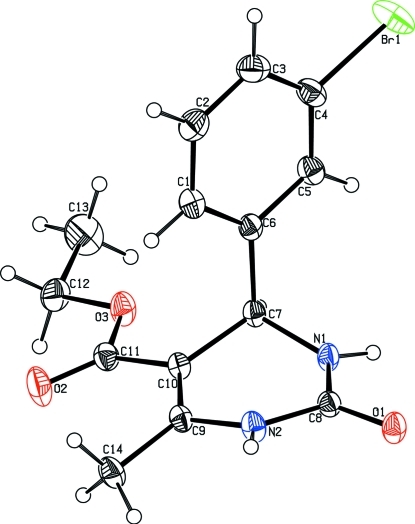
Perspective view of the molecule showing the thermal ellipsoids drawn at 30% probability level.

**Fig. 2.**
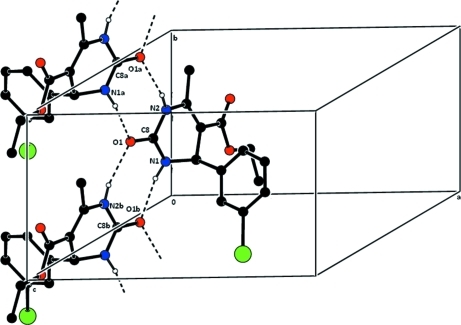
The crystal packing of the molecules viewed along c axis. For clarity, hydrogen atoms which are not involved in hydrogen bonding are omitted

## supplementary crystallographic information

### Comment

In recent years, acid-catalyzed cyclocondensation of β-ketoesters with aromatic aldehydes and ureas, known as the Biginelli reaction, has attracted remarkable attention. The resulting dihydropyrimidinones (DHPM) have drawn wide-spread interest due to their broad range of therapeutic and pharmacological properties (Kappe, 2000). Owing to this background and in order to obtain detailed information on its molecular conformation, the *x*-ray structure of the title compound has been determined and is discussed here.

The *ORTEP* plot of the title molecule is shown in Fig.1. In the present structure dihydropyrimidinone ring adopts a boat conformation with atoms N2 and C7 deviating by 0.159 (2) and 0.214 (2) Å, respectively from the least square plane defined by the remaining atoms N1/C8/C9/C10 in the ring.

The puckering parameters (Cremer & Pople, 1975) are Q = 0.339 (2) Å;Θ = 74.9 (3)° and φ = 50.2 (3)°. Atom Br1 deviates from the plane of the C1—C6 benzene ring by -0.024 (1) Å. The ethyl acetate group shows an extended conformation [C11—O3—C12—C13] = 174.7 (2)°. In the crystal structure, the molecules at (*x*, *y*, *z*), -*x*,-1/2 + *y*,1/2 - *z*,and -*x*,1/2 + *y*,1/2 - *z* are linked by N(1)—H(1 A)···O(1) and N(2)—H(2 A)···O(1) hydrogen bonds and forming a ring motif *R_2_^2^*(8) and generating a one dimensional chain extending in [010] direction.

### Experimental

A mixture of ethylacetoacetate (5 mmol), 3-bromobenzaldehyde (5 mmol) and urea (6 mmol) was refluxed in ethanol in the presence of concentrated hydrochloric acid as catalyst. After the completion of reaction, it was quenched in ice cold water and the obtained precipitate was filtered, dried and crystallized from ethanol to obtain the title compound.

### Refinement

All H atoms were positioned geometrically (N—H = 0.86 Å, C—H = 0.93–0.98 Å) and allowed to ride on their parent atoms, with 1.5*U*_eq_(C) for methyl H and 1.2 *U*_eq_(C,N) for other H atoms.

### Crystal data

**Table:**

C_14_H_15_BrN_2_O_3_	*F*(000) = 688
*M**_r_* = 339.19	*D*_x_ = 1.588 Mg m^−^^3^
Monoclinic, *P*2_1_/*c*	Mo *K*α radiation, λ = 0.71073 Å
Hall symbol: -P 2ybc	Cell parameters from 754 reflections
*a* = 12.5184 (11) Å	θ = 1.8–28.4°
*b* = 7.3412 (5) Å	µ = 2.91 mm^−^^1^
*c* = 17.0426 (15) Å	*T* = 293 K
β = 115.086 (6)°	Block, colourless
*V* = 1418.5 (2) Å^3^	0.25 × 0.23 × 0.2 mm
*Z* = 4	

### Data collection

**Table:**

Bruker SMART APEXII area-detector diffractometer	3541 independent reflections
Radiation source: fine-focus sealed tube	2597 reflections with *I* > 2σ(*I*)
graphite	*R*_int_ = 0.033
ω and φ scans	θ_max_ = 28.4°, θ_min_ = 1.8°
Absorption correction: multi-scan (*SADABS*; Bruker, 2008)	*h* = −16→14
*T*_min_ = 0.488, *T*_max_ = 0.559	*k* = −9→9
13419 measured reflections	*l* = −22→22

### Refinement

**Table:**

Refinement on *F*^2^	Primary atom site location: structure-invariant direct methods
Least-squares matrix: full	Secondary atom site location: difference Fourier map
*R*[*F*^2^ > 2σ(*F*^2^)] = 0.034	Hydrogen site location: inferred from neighbouring sites
*wR*(*F*^2^) = 0.092	H-atom parameters constrained
*S* = 1.02	*w* = 1/[σ^2^(*F*_o_^2^) + (0.0446*P*)^2^ + 0.4545*P*] where *P* = (*F*_o_^2^ + 2*F*_c_^2^)/3
3541 reflections	(Δ/σ)_max_ = 0.001
183 parameters	Δρ_max_ = 0.32 e Å^−^^3^
0 restraints	Δρ_min_ = −0.57 e Å^−^^3^

### Special details

**Table:**

Geometry. All e.s.d.'s (except the e.s.d. in the dihedral angle between two l.s. planes) are estimated using the full covariance matrix. The cell e.s.d.'s are taken into account individually in the estimation of e.s.d.'s in distances, angles and torsion angles; correlations between e.s.d.'s in cell parameters are only used when they are defined by crystal symmetry. An approximate (isotropic) treatment of cell e.s.d.'s is used for estimating e.s.d.'s involving l.s. planes.
Refinement. Refinement of *F*^2^ against ALL reflections. The weighted *R*-factor *wR* and goodness of fit *S* are based on *F*^2^, conventional *R*-factors *R* are based on *F*, with *F* set to zero for negative *F*^2^. The threshold expression of *F*^2^ > σ(*F*^2^) is used only for calculating *R*-factors(gt) *etc*. and is not relevant to the choice of reflections for refinement. *R*-factors based on *F*^2^ are statistically about twice as large as those based on *F*, and *R*- factors based on ALL data will be even larger.

### Fractional atomic coordinates and isotropic or equivalent isotropic displacement parameters (Å^2^)

**Table:**

	*x*	*y*	*z*	*U*_iso_*/*U*_eq_	
Br1	0.44441 (3)	−0.16428 (4)	0.393184 (18)	0.06851 (13)	
O1	−0.01129 (13)	0.45992 (18)	0.25987 (9)	0.0366 (3)	
O2	0.19555 (17)	0.5721 (2)	−0.01580 (10)	0.0542 (4)	
O3	0.20747 (13)	0.2739 (2)	0.01176 (9)	0.0398 (3)	
N1	0.07460 (14)	0.3000 (2)	0.18856 (10)	0.0300 (3)	
H1A	0.0413	0.2007	0.1932	0.036*	
N2	0.07540 (15)	0.6114 (2)	0.18553 (10)	0.0328 (4)	
H2A	0.0724	0.7129	0.2097	0.039*	
C1	0.37388 (19)	0.3902 (3)	0.25000 (14)	0.0394 (5)	
H1	0.3602	0.5013	0.2212	0.047*	
C2	0.4823 (2)	0.3559 (3)	0.31773 (15)	0.0471 (5)	
H2	0.5408	0.4446	0.3338	0.056*	
C3	0.5056 (2)	0.1925 (3)	0.36196 (14)	0.0455 (5)	
H3	0.5785	0.1701	0.4077	0.055*	
C4	0.41681 (19)	0.0634 (3)	0.33589 (13)	0.0390 (5)	
C5	0.30810 (18)	0.0945 (3)	0.26915 (12)	0.0349 (4)	
H5	0.2498	0.0056	0.2536	0.042*	
C6	0.28529 (16)	0.2598 (3)	0.22473 (11)	0.0287 (4)	
C7	0.16221 (16)	0.2905 (2)	0.15251 (11)	0.0271 (4)	
H7	0.1428	0.1848	0.1138	0.033*	
C8	0.04431 (16)	0.4545 (2)	0.21425 (11)	0.0285 (4)	
C9	0.11175 (17)	0.6132 (2)	0.11857 (11)	0.0293 (4)	
C10	0.15120 (16)	0.4584 (2)	0.09844 (11)	0.0281 (4)	
C11	0.18583 (17)	0.4468 (3)	0.02604 (11)	0.0329 (4)	
C12	0.2432 (2)	0.2459 (4)	−0.05813 (13)	0.0446 (5)	
H12A	0.3109	0.3214	−0.0493	0.054*	
H12B	0.1793	0.2784	−0.1132	0.054*	
C13	0.2736 (3)	0.0503 (4)	−0.05805 (18)	0.0688 (8)	
H13A	0.3368	0.0196	−0.0033	0.103*	
H13B	0.2979	0.0279	−0.1036	0.103*	
H13C	0.2059	−0.0231	−0.0672	0.103*	
C14	0.1005 (2)	0.7953 (3)	0.07619 (13)	0.0397 (5)	
H14A	0.1762	0.8535	0.0982	0.060*	
H14B	0.0463	0.8696	0.0885	0.060*	
H14C	0.0716	0.7794	0.0147	0.060*	

### Atomic displacement parameters (Å^2^)

**Table:**

	*U*^11^	*U*^22^	*U*^33^	*U*^12^	*U*^13^	*U*^23^
Br1	0.0843 (2)	0.04469 (16)	0.06112 (18)	0.01772 (13)	0.01586 (15)	0.02071 (12)
O1	0.0494 (8)	0.0265 (7)	0.0486 (8)	0.0023 (6)	0.0348 (7)	0.0010 (6)
O2	0.0830 (12)	0.0447 (10)	0.0545 (9)	0.0032 (8)	0.0483 (9)	0.0133 (8)
O3	0.0568 (9)	0.0381 (8)	0.0374 (7)	−0.0002 (7)	0.0322 (7)	−0.0014 (6)
N1	0.0377 (8)	0.0216 (7)	0.0397 (8)	−0.0004 (6)	0.0250 (7)	0.0030 (6)
N2	0.0496 (10)	0.0197 (7)	0.0374 (8)	−0.0007 (6)	0.0267 (8)	−0.0020 (6)
C1	0.0426 (11)	0.0347 (10)	0.0429 (11)	−0.0015 (9)	0.0202 (9)	0.0064 (9)
C2	0.0382 (11)	0.0504 (14)	0.0514 (12)	−0.0075 (10)	0.0178 (10)	0.0000 (10)
C3	0.0401 (11)	0.0548 (14)	0.0389 (11)	0.0097 (10)	0.0140 (9)	0.0021 (10)
C4	0.0519 (12)	0.0331 (11)	0.0352 (9)	0.0121 (9)	0.0216 (9)	0.0059 (8)
C5	0.0441 (11)	0.0278 (9)	0.0354 (9)	0.0015 (8)	0.0195 (9)	−0.0002 (8)
C6	0.0362 (9)	0.0273 (9)	0.0291 (8)	0.0036 (7)	0.0202 (8)	0.0001 (7)
C7	0.0356 (9)	0.0219 (8)	0.0289 (8)	−0.0002 (7)	0.0186 (7)	−0.0004 (7)
C8	0.0327 (9)	0.0247 (9)	0.0312 (8)	0.0001 (7)	0.0165 (8)	0.0016 (7)
C9	0.0334 (9)	0.0258 (9)	0.0289 (8)	−0.0037 (7)	0.0133 (7)	0.0021 (7)
C10	0.0330 (9)	0.0269 (9)	0.0261 (8)	−0.0017 (7)	0.0142 (7)	0.0019 (7)
C11	0.0358 (10)	0.0356 (10)	0.0284 (8)	−0.0012 (8)	0.0146 (8)	0.0015 (8)
C12	0.0493 (12)	0.0619 (15)	0.0323 (10)	0.0057 (11)	0.0266 (9)	−0.0014 (10)
C13	0.099 (2)	0.0677 (19)	0.0591 (15)	0.0209 (16)	0.0522 (16)	−0.0020 (14)
C14	0.0533 (12)	0.0255 (9)	0.0433 (11)	0.0000 (8)	0.0235 (10)	0.0065 (8)

### Geometric parameters (Å, °)

**Table:**

Br1—C4	1.892 (2)	C4—C5	1.373 (3)
O1—C8	1.245 (2)	C5—C6	1.394 (3)
O2—C11	1.202 (2)	C5—H5	0.9300
O3—C11	1.341 (2)	C6—C7	1.527 (3)
O3—C12	1.453 (2)	C7—C10	1.510 (2)
N1—C8	1.328 (2)	C7—H7	0.9800
N1—C7	1.469 (2)	C9—C10	1.340 (3)
N1—H1A	0.8600	C9—C14	1.497 (3)
N2—C8	1.371 (2)	C10—C11	1.474 (2)
N2—C9	1.396 (2)	C12—C13	1.486 (4)
N2—H2A	0.8600	C12—H12A	0.9700
C1—C2	1.382 (3)	C12—H12B	0.9700
C1—C6	1.388 (3)	C13—H13A	0.9600
C1—H1	0.9300	C13—H13B	0.9600
C2—C3	1.380 (3)	C13—H13C	0.9600
C2—H2	0.9300	C14—H14A	0.9600
C3—C4	1.383 (3)	C14—H14B	0.9600
C3—H3	0.9300	C14—H14C	0.9600
			
C11—O3—C12	116.02 (16)	O1—C8—N1	123.16 (16)
C8—N1—C7	123.15 (15)	O1—C8—N2	120.97 (16)
C8—N1—H1A	118.4	N1—C8—N2	115.85 (16)
C7—N1—H1A	118.4	C10—C9—N2	118.96 (16)
C8—N2—C9	122.68 (15)	C10—C9—C14	127.08 (17)
C8—N2—H2A	118.7	N2—C9—C14	113.95 (17)
C9—N2—H2A	118.7	C9—C10—C11	122.34 (16)
C2—C1—C6	120.4 (2)	C9—C10—C7	118.95 (15)
C2—C1—H1	119.8	C11—C10—C7	118.71 (16)
C6—C1—H1	119.8	O2—C11—O3	122.56 (17)
C3—C2—C1	121.3 (2)	O2—C11—C10	126.29 (19)
C3—C2—H2	119.3	O3—C11—C10	111.15 (16)
C1—C2—H2	119.3	O3—C12—C13	107.72 (19)
C2—C3—C4	117.7 (2)	O3—C12—H12A	110.2
C2—C3—H3	121.1	C13—C12—H12A	110.2
C4—C3—H3	121.1	O3—C12—H12B	110.2
C5—C4—C3	122.09 (19)	C13—C12—H12B	110.2
C5—C4—Br1	118.33 (17)	H12A—C12—H12B	108.5
C3—C4—Br1	119.58 (16)	C12—C13—H13A	109.5
C4—C5—C6	119.84 (19)	C12—C13—H13B	109.5
C4—C5—H5	120.1	H13A—C13—H13B	109.5
C6—C5—H5	120.1	C12—C13—H13C	109.5
C1—C6—C5	118.62 (18)	H13A—C13—H13C	109.5
C1—C6—C7	123.28 (17)	H13B—C13—H13C	109.5
C5—C6—C7	118.07 (17)	C9—C14—H14A	109.5
N1—C7—C10	109.00 (14)	C9—C14—H14B	109.5
N1—C7—C6	110.31 (14)	H14A—C14—H14B	109.5
C10—C7—C6	114.35 (15)	C9—C14—H14C	109.5
N1—C7—H7	107.6	H14A—C14—H14C	109.5
C10—C7—H7	107.6	H14B—C14—H14C	109.5
C6—C7—H7	107.6		
			
C6—C1—C2—C3	−0.1 (3)	C9—N2—C8—N1	14.2 (3)
C1—C2—C3—C4	0.4 (4)	C8—N2—C9—C10	−20.2 (3)
C2—C3—C4—C5	−0.8 (3)	C8—N2—C9—C14	159.42 (18)
C2—C3—C4—Br1	179.20 (17)	N2—C9—C10—C11	177.02 (16)
C3—C4—C5—C6	0.8 (3)	C14—C9—C10—C11	−2.5 (3)
Br1—C4—C5—C6	−179.11 (14)	N2—C9—C10—C7	−3.8 (3)
C2—C1—C6—C5	0.1 (3)	C14—C9—C10—C7	176.64 (18)
C2—C1—C6—C7	177.96 (19)	N1—C7—C10—C9	28.6 (2)
C4—C5—C6—C1	−0.5 (3)	C6—C7—C10—C9	−95.4 (2)
C4—C5—C6—C7	−178.46 (17)	N1—C7—C10—C11	−152.22 (15)
C8—N1—C7—C10	−36.2 (2)	C6—C7—C10—C11	83.8 (2)
C8—N1—C7—C6	90.2 (2)	C12—O3—C11—O2	−0.3 (3)
C1—C6—C7—N1	−111.5 (2)	C12—O3—C11—C10	−179.66 (16)
C5—C6—C7—N1	66.4 (2)	C9—C10—C11—O2	8.7 (3)
C1—C6—C7—C10	11.8 (2)	C7—C10—C11—O2	−170.5 (2)
C5—C6—C7—C10	−170.39 (16)	C9—C10—C11—O3	−171.96 (17)
C7—N1—C8—O1	−165.42 (17)	C7—C10—C11—O3	8.9 (2)
C7—N1—C8—N2	16.2 (2)	C11—O3—C12—C13	174.7 (2)
C9—N2—C8—O1	−164.23 (17)		

### Hydrogen-bond geometry (Å, °)

**Table:**

*D*—H···*A*	*D*—H	H···*A*	*D*···*A*	*D*—H···*A*
N1—H1A···O1^i^	0.86	2.04	2.868 (2)	161
N2—H2A···O1^ii^	0.86	2.12	2.948 (2)	162

Symmetry codes: (i) −*x*, *y*−1/2, −*z*+1/2; (ii) −*x*, *y*+1/2, −*z*+1/2.

## References

[bb1] Atwal, K. S. (1990). *J. Med. Chem* **33**, 1510–1515. 10.1021/jm00167a035 2329573

[bb2] Bernstein, J., Davis, R. E., Shimoni, L. & Chang, N.-L. (1995). *Angew. Chem. Int. Ed. Engl.* **34**, 1555–1573.

[bb3] Biginelli, P. (1891). *Ber. Dtsch Chem. Ges.* **24**, 2962–2965.

[bb4] Bruker (2008). *APEX2*, *SAINT* and *SADABS* Bruker AXS Inc., Madison, Wisconsin, USA.

[bb5] Cremer, D. & Pople, J. A. (1975). *J. Am. Chem. Soc.* **97**, 1354–1358.

[bb6] Farrugia, L. J. (1997). *J. Appl. Cryst.* **30**, 565.

[bb7] Fun, H.-K., Yeap, C. S., Babu, M. & Kalluraya, B. (2009). *Acta Cryst.* E**65**, o1188–o1189. 10.1107/S1600536809015918 PMC2969791 21583061

[bb8] Kappe, C. O. (2000). *Eur. J. Med. Chem.* **35**, 1043–1052. 10.1016/s0223-5234(00)01189-2 11248403

[bb9] Sheldrick, G. M. (2008). *Acta Cryst.* A**64**, 112–122. 10.1107/S0108767307043930 18156677

[bb10] Spek, A. L. (2009). *Acta Cryst.* D**65**, 148–155. 10.1107/S090744490804362X PMC2631630 19171970

